# Editorial: Meningioma: From basic research to clinical translational study, volume II

**DOI:** 10.3389/fonc.2023.1150514

**Published:** 2023-03-27

**Authors:** Hailiang Tang, Zhewei Shen, David D. Eisenstat, Ian F. Dunn

**Affiliations:** ^1^Department of Neurosurgery, Huashan Hospital, Shanghai Medical College, Fudan University, Shanghai, China; ^2^National Center for Neurological Disorders, Shanghai, China; ^3^Shanghai Key Laboratory of Brain Function and Restoration and Neural Regeneration, Shanghai, China; ^4^Neurosugical Institute of Fudan University, Shanghai, China; ^5^Shanghai Clinical Medical Center of Neurosurgery, Shanghai, China; ^6^Department of Paediatrics, University of Melbourne, Parkville, VIC, Australia; ^7^Murdoch Children’s Research Institute, The Royal Children’s Hospital Melbourne, Parkville, VIC, Australia; ^8^Department of Neurosurgery, University of Oklahoma Health Sciences Center, Oklahoma City, OK, United States

**Keywords:** meningioma, molecular alteration, genetic mutation, cancer stem cells, targeted therapy

The last Research Topic “*Meningioma: From Basic Research to Clinical Translational Study*” published in ***Frontiers in Oncology*** (1), featured more than 40 papers on meningiomas discussing aspects of basic research, clinical management, and adjuvant therapies (1). This time, in the 31 manuscripts included in Research Topic II, papers mainly covered advances in molecular genetics of meningiomas and targeted therapies for these tumors.

## 1 Molecular genetic alterations in meningiomas

Meningiomas are thought to arise from the meninges and many studies are currently focused on meningeal tumorigenesis mechanisms (2, 3). The landmark 2021 World Health Organization (WHO) edition of the classification of central nervous system (CNS) tumors endorsed, for the first time, molecular grading schemes for meningiomas with tumor classification based on genetic/epigenetic alterations (4–6), including methylation patterns, copy number alterations and driver mutations, such as TERT promoter mutation, and homozygous CDKN2A/B deletion in anaplastic meningiomas. Deng et al. found that *TERT* alterations were strongly associated with tumor progression and poor outcome of *de novo* high-grade meningiomas patients after postoperative adjuvant radiotherapy.

In addition, several reviews included in this Research Topic highlighted our current understanding of the molecular and genetic alterations in meningiomas. Peng et al. suggested that thorough understanding of these changes will assist in identifying “high-risk” factors for recurrent and progressive meningiomas with a view towards establishing personalized and precise therapy for patients with meningioma. Wang et al. discussed preclinical and clinical evidence of molecular classification schemes for meningioma prognostication. Kim et al. explored the clinical significance of next-generation sequencing in characterizing the molecular profiles of high-grade meningiomas.

There is accumulating evidence that the WHO grade alone may not provide an adequate prediction of meningioma behavior (6). In their study, Roehrkasse et al. proposed the integration of routine molecular profiling with histopathologic grading to guide clinical decision-making strategies in patients with meningiomas. Moreover, Chen et al. suggested that the N6-methylation (m6A) regulators segregated meningiomas into two distinct m6A clusters, which correlated with different m6A regulator gene expression and immune cell infiltration.

## 2 Translational therapies for meningiomas

Molecular data can inform future directions in therapeutic strategies for meningioma. Currently, multiple targetable genetic alterations have been identified in meningiomas, and targeted therapies (including focal adhesion kinase inhibitor GSK2256098 and CDK4/6 inhibitors) are being clinically evaluated in meningioma patients (7, 8). Lynes et al. discussed molecular classification schemes for meningiomas, and reviewed current multi-targeted therapies for meningiomas. Young et al. systematically described the preclinical evidence for CDK4/6 inhibitors as therapies for high-grade meningiomas, and summarized clinical trials with these inhibitors. Patel et al. comprehensively summarized their experience regarding three cases of progressive meningiomas, and discussed targeted drug treatment for aggressive and recurrent meningiomas.

Along with the advances in molecular characterization of meningiomas, there has been recent progress in understanding the immune profile of meningiomas. The meningioma immune microenvironment mostly comprises macrophages, T cells and mast cells. Kannapadi et al. in their excellent review described the immune signatures of meningiomas and discussed the interactions between molecular patterns and immune signatures in meningiomas. Moreover, they detailed several clinical trials using immunotherapy in meningiomas. Programmed death-ligand 1 (PD-L1) is one of the most frequently studied immune checkpoint molecules in meningiomas, and clinical trials on PD-1 blockade have been reported (9, 10). Furthermore, in the cellular and rodent model level, Deng et al. demonstrated that the expression of nicotinamide phosphoribosyltransferase (NAMPT) is upregulated in anaplastic meningiomas, and that the NAMPT inhibitor-FK866 can significantly suppress the growth of anaplastic meningiomas. Moreover, FK866 can inhibit PD-L1 expression in anaplastic meningiomas.

## 3 Radiomics enabled prediction models for meningiomas

Histopathological grading alone is insufficient to achieve optimal risk stratification. In fact, it is clinically difficult to predict postoperative recurrence and guide individual treatment decisions only by tumor classification. In this Research Topic collection, several papers using radiological techniques for meningioma prediction are included. Zhang et al. in their study generated an MRI-based prediction model of meningioma recurrence after surgery, which was coupled with clinical prognostic factors and histopathological grades. Chen et al. established an integrated model based on clinical, radiological and pathological factors to predict the postoperative recurrence of atypical meningioma. Sun et al. used a combination of radiomics analysis and machine learning, showing clinical utility in the prediction of preoperative NF2 status in meningiomas. Li et al. constructed a model for predicting brain invasion in WHO grade II meningioma by using preoperative MRI. Takase et al. assumed that bone invasion may be a preoperative predictor of the extent of surgical resection for meningiomas.

Interestingly, Roytman et al. used combined PET/MRI to demonstrate a significant correlation between tumor vascularity and somatostatin receptor-2 (SSTR2) expression in WHO II/III, but not in WHO I meningiomas, suggesting biological differences in the relationship between tumor vascularity and SSTR2 expression in higher-grade meningiomas. Chen et al. differentiated intracranial hemangiopericytoma/solitary fibrous tumor (HPC/SFT) and meningioma *via* deep learning approaches based on preoperative MRI. HPC/SFT have similar radiological characteristics as meningioma, but with different clinical management and outcomes. In addition, Fan et al. also used a clinical-radiomic model to preoperatively distinguish HPC and angiomatous meningioma.

## 4 Clinical management for meningiomas

Meningiomas are very common brain tumors, and diverse in intracranial locations and behavior. In this Research Topic, several papers discussing localization features and clinical therapies for meningiomas were also collected. In the parasagittal meningiomas derived from arachnoidal cap cells distributed in the arachnoid granulations, Ye et al. used anatomical and histological techniques to reveal the different anatomical types of arachnoid granulations. Based on these features, they speculated on the different growth patterns of parasagittal meningioma, which can guide the neurosurgeon to remove the tumor safely. Yamada et al. identified factors predictive of clinical symptoms in patients with convexity, parasagittal and falx meningiomas, which may be useful in improving management of patients. Mederer et al. showed that surgical resection leads to long-term improvement of neurological impairment in the majority of patients with non-skull base meningiomas. However, tumor location, biology and extent of resection are essential factors influencing neurological outcome.

Unlike more superficially based tumors, meningiomas deeply located inside the skull and adjacent to critical neurovascular structures are more challenging to access and more difficult to resect. Gao et al. concluded that the optimal surgical approach for petroclival meningiomas (PCMs) depends on the size, extension of the tumor and the anatomical relationship between the tumor and the cranial nerves. Ding et al. showed combined microscopic-endoscopic surgery for pineal region meningiomas eliminates microscopic blind spots, thus compensating for the shortcomings of the traditional occipital transtentorial approach. Liu et al. introduced their experiences in the management of falcotentorial junction tumors, and concluded that the surgical approach selection depends on the growth characteristics of the tumor and venous or sinus involvement.

For large meningiomas, adjuvant treatments may also be used. Yin et al. showed that preoperative embolization can significantly lower surgical complications and long-term disabilities for meningioma patients. Gong et al. investigated the efficiency and safety of dose-staged Gamma Knife radiosurgery as an alternative option for large volume meningiomas adjacent to critical structures. Consensus regarding the need for adjuvant radiotherapy in patients with atypical meningiomas has not been reached. Song et al demonstrated that regardless of either gross total resection or subtotal resection of the tumor, postoperative radiotherapy improved progression-free survival and overall survival for patients.

## 5 Conclusions

Meningioma recurrence is related to multiple factors, including age, extent of surgical excision, and histological grade as the currently accepted surrogate for our understanding of tumor behavior, though this is challenged by our understanding of genomics and epigenomics. Recently, an increasing number of studies have reported that the presence of cancer stem cells (CSCs) within meningioma is closely associated with tumor aggressiveness, recurrence and therapy resistance (11, 12). Furthermore, patient-derived xenograft (PDX) models using patient-derived tumor cells transplanted into immunodeficient mice have also emerged as important tools for translational research in meningiomas (13).

## Author contributions

HT wrote the manuscript, ZS collected the references, and DE and ID revised the manuscript. All authors contributed to the article and approved the submitted version.
